# Zebrafish Models of Prader-Willi Syndrome: Fast Track to Pharmacotherapeutics

**DOI:** 10.3390/diseases4010013

**Published:** 2016-03-08

**Authors:** Emma D. Spikol, Caroline E. Laverriere, Maya Robnett, Gabriela Carter, Erin Wolfe, Eric Glasgow

**Affiliations:** Department of Oncology, Georgetown University Medical Center, 4000 Reservoir Road, N.W., Washington, DC 20057, USA; eds46@georgetown.edu (E.D.S.); celaverriere@gmail.com (C.E.L.); robnett.maya@gmail.com (M.R.); gcarter086@gmail.com (G.C.); emw81@georgetown.edu (E.W.)

**Keywords:** neuroendocrine, oxytocin, zebrafish

## Abstract

Prader-Willi syndrome (PWS) is a rare genetic neurodevelopmental disorder characterized by an insatiable appetite, leading to chronic overeating and obesity. Additional features include short stature, intellectual disability, behavioral problems and incomplete sexual development. Although significant progress has been made in understanding the genetic basis of PWS, the mechanisms underlying the pathogenesis of the disorder remain poorly understood. Treatment for PWS consists mainly of palliative therapies; curative therapies are sorely needed. Zebrafish, *Danio rerio*, represent a promising way forward for elucidating physiological problems such as obesity and identifying new pharmacotherapeutic options for PWS. Over the last decade, an increased appreciation for the highly conserved biology among vertebrates and the ability to perform high-throughput drug screening has seen an explosion in the use of zebrafish for disease modeling and drug discovery. Here, we review recent advances in developing zebrafish models of human disease. Aspects of zebrafish genetics and physiology that are relevant to PWS will be discussed, and the advantages and disadvantages of zebrafish models will be contrasted with current animal models for this syndrome. Finally, we will present a paradigm for drug screening in zebrafish that is potentially the fastest route for identifying and delivering curative pharmacotherapies to PWS patients.

## 1. Introduction

Prader-Willi syndrome (PWS) is a complex neurogenetic disorder, which results in cognitive and neuroendocrine dysfunction. Individuals with PWS present a spectrum of clinically observed phenotypes. Possibly the most debilitating symptom is insatiable appetite, which often leads to chronic overeating and obesity. Between the ages of 4.5 and 8, children with PWS experience rapid onset of hyperphagia and obesity [1,2,3]. While behavioral therapy can mitigate weight gain, complications from obesity are the leading cause of morbidity in PWS [4]. Patients also suffer from growth hormone (GH) deficiency resulting in decreased growth velocity and short stature if untreated. GH treatment during childhood allows patients to reach full height [5]. Older children and adults have hypoplastic genitalia and dysmorphic features including small hands and feet, flat surface of the ulnar side of hands and inner legs, a narrow forehead, almond-shaped eyes and a downturned mouth. Sleep-disordered breathing and daytime hypersomnolence are also associated with PWS. PWS individuals are cognitively disabled, with an average IQ of 65 [1]. Finally, the disorder causes behavioral abnormalities such as skin picking and tantrums. Comorbidity with neuropsychiatric disorders is high. Current treatment options for PWS consist of hormone administration and behavioral therapy (Table 1) [1,4,6,7].

The genetic basis of PWS is an alteration in the paternally inherited allele of the imprinted chromosome 15q11–q13 region. Although it remains unclear how genetic defects at 15q11–q13 result in the clinical manifestations of PWS, it is likely that hypothalamic dysfunction underlies many of these symptoms [4]. Studies of PWS biology are likely to bring about a deeper understanding of the molecular, cellular, physiological and behavioral basis of this syndrome, and eventually bring targeted, curative therapies to PWS patients. Already, several mouse models of PWS have been created, and despite failure to recapitulate all aspects of PWS, they have proved very informative [6]. Several mouse models, particularly those with inactivated genes implicated in PWS-like disorders, support hypothalamic dysfunction as a major feature of PWS. However, notwithstanding the utility of mouse models for understanding the pathobiology of PWS, mice are ill-suited for high-throughput screens. 

Over the last few decades, zebrafish have emerged as an excellent model to study vertebrate development and human disease. Zebrafish offer many advantages over other vertebrate species. In particular, their fecundity and small adult body size allows them to be housed at high densities in a laboratory setting. Furthermore, zebrafish reach sexual maturity at three months, akin to mice, but embryonic development occurs more rapidly than in mice with most zebrafish organs and glands developed by five days post-fertilization. As vertebrates, zebrafish have many of the same genes, cells, tissues, glands, organs systems and body plans as their mammalian counterparts [8,9]. Moreover, although they lack much of the highly developed mammalian cortical brain regions, evolutionarily older structures including the neuroendocrine system are remarkably conserved. Zebrafish embryos are capable of fitting into 96-well plates, in addition to their relative translucency, making them ideal candidates for high-throughput screens and advanced imaging techniques. Finally, the zebrafish genome-sequencing project, which began in 2001, found that approximately 70% of human genes have at least one zebrafish ortholog with 82% of known human disease genes having a zebrafish ortholog [10]. 

**Figure 1:**
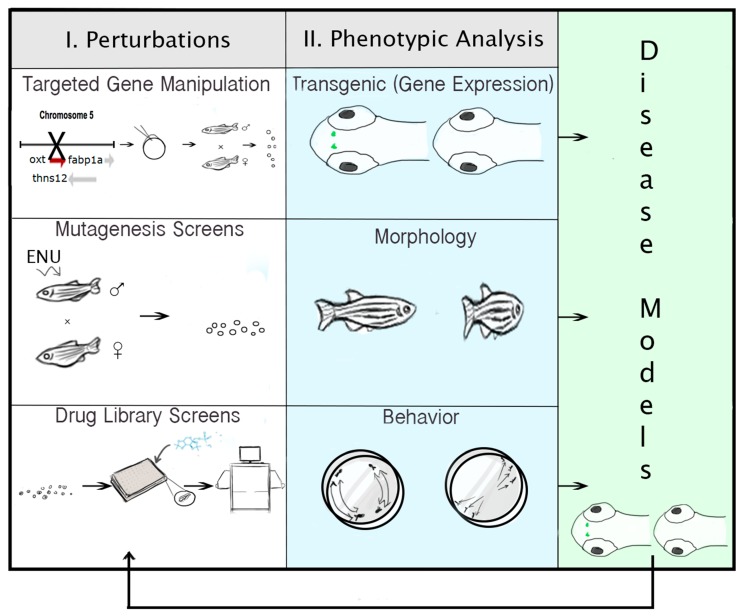
Overview of approaches for investigating PWS using zebrafish. (**I**) Genes and molecular pathways can be genetically or pharmacologically perturbed. (**II**) The effect of perturbations can be evaluated using a variety of phenotypic analyses. PWS endophenotypes may then be utilized as disease models and subjected to drug screening to identify potential pharmacotherapeutics.

Here, we summarize progress in understanding PWS pathobiology in light of creating zebrafish models for this syndrome. We discuss three investigative strategies commonly used in zebrafish research, including: (1) the production of gene-knockout models paired with high-throughput screens to pinpoint drug targets; (2) the use of large scale mutagenesis screens to identify and investigate endophenotypes that are relevant to PWS; and (3) the use of high throughput drug screens to identify compounds with activity specific to neuronal populations and behaviors affected by PWS (Figure 1). This last strategy may provide the fastest route for identifying curative pharmacotherapeutic compounds when used to screen libraries of FDA approved drugs.

## 2. Discussion

### 2.1. Genetics

The genetic basis of PWS is the loss of imprinted genes in the 15q11–q13 region, which are exclusively expressed from the paternal chromosome. Loss of the paternally expressed genes associated with PWS can occur in multiple ways. In about two thirds of PWS cases, there is a deletion of 4–4.5 Mb in the paternal copy of the 15q11–q13 region. In about 25% of cases, the cause is uniparental disomy resulting in the inheritance of two copies of chromosome 15 from the mother. In fewer than 3% of cases, PWS is caused by a submicroscopic deletion or epimutation of a sequence known as the imprinting center (IC). Finally, rare cases result from chromosomal translocations or rearrangements [1]. Imprinted PWS-associated genes located in the 15q11–q13 region are subject to varying degrees of conservation in mouse and zebrafish. In mouse, chromosome 7 B/C is a syntenic imprinted region containing several orthologs of PWS-associated genes. The imprinted PWS gene cluster is absent in fish, likely arising in the mammalian lineage. Nonetheless, several genes have putative orthologs in zebrafish, including makorin ring finger protein 3 (*MKRN3*); MAGE family member L2 (*MAGEL2*); necdin, MAGE family member (*NDN*); and small nuclear ribonucleoprotein polypeptide N (*SNRPN*) (Table 2).

*MKRN3*, along with *MKRN1,* *MKRN2*, and *MKRN4,* is a member of a unique family of ring finger containing proteins with ubiquitin ligase and transcriptional coregulatory functions. In mice, *Mkrn3* is highly expressed in the fetal arcuate nucleus, and in humans, *MKRN3* mutations have been implicated in precocious puberty, thus implicating this gene in hypothalamic function. *MKRN3* in mammals likely arose by retrotransposition of the paralogous *MKRN1* gene [12,13]. Therefore, zebrafish lack the *mkrn3* gene, with the most similar gene being *mkrn1*. Unlike the other zebrafish orthologs for PWS-associated genes, this ancestral gene has extensive synteny with both mouse and human [14].

*MAGEL2* and *NDN* code for MAGE proteins, a large family of poorly understood ubiquitin ligase proteins. The MAGE family is unusual in mammals in that there have been multiple species specific expansions of various MAGE family members, with over 50 genes in humans. In contrast, non-mammalian species such as zebrafish have a single representative of the MAGE family. Thus, in zebrafish, the singular MAGE family gene, *ndnl2*, is most similar to both *MAGEL2* and *NDN* [15].

*SNRPN* encodes a bicistronic transcript. One product, Snrpn Upstream Reading Frame (SNURF), is a non-coding RNA of unknown function. The other encodes SmN, a LSm family protein, which is thought to be involved in pre-mRNA processing. The *SNRPN* gene also contains a sequence known as the imprinting center (IC), which is required for silencing the maternal allele [16]. Zebrafish do not have a *snrpn* gene, they do however have an ortholog of *SNRPB*, which likely gave rise to *SNRPN* by duplication in the mammalian lineage [12]. The amino acid sequence conservation among zebrafish *snrpb* and human SNRPN very high (93%; Table 2), especially in the LSm superfamily conserved domain, which is involved in RNA processing. 

There are some differences in the imprinted PWS region between mice and humans. In mice, an imprinted gene known as *Peg12* lies within the PWS region. *Peg12* is a member of the glycogen synthase kinase 3 binding protein (Gbp) family, with presumed functions in the regulation of Wnt signaling. The most closely related human gene, Frequently Rearranged In Advanced T-Cell Lymphomas 1 (*FRAT1*), is located on chromosome 10, while the orthologous mouse *Frat1* gene is located on chromosome 19 and is not known to be imprinted. Knockout of either *peg12* or *Frat1* had no phenotype so the physiological role of these genes is unclear. In zebrafish, only a single ancestral gene, *gbp*, is present and, although a mutant exists, it has not yet been characterized.

An additional difference is that in humans, but not mice, the nuclear pore-associated protein *NPAP1* gene is found within the imprinted PWS region. *NPAP1* likely arose in primates after several rounds of retrotranspostion of the ancestral *POM121* gene [17]. Thus, like mice, zebrafish do not have an *npap1* gene, however, they both do have the ancestral *pom121* gene. Neither mouse nor zebrafish *pom121* mutants have been characterized.

In addition to the protein coding genes discussed above, the PWS-region contains multiple gene clusters, called SNORDs, encoding small nucleolar RNAs (snoRNAs) belonging to the C/D box group [18,19]. For the *SNORD107*, *SNORD64*, *SNORD115* and *SNORD116* clusters, orthologous groups exist in mouse, although copy numbers vary. However, no mouse orthologs have been identified for *SNORD108*, *SNORD109A* or *SNORD109B* (Table 2). Although SnoRNA genes are typically conserved sequences, those in the PWS region belong to a group of eutherian-specific imprinted SnoRNAs for which homologs in non-eutherian vertebrates have yet to be identified [11]. While many SnoRNA genes can be found in zebrafish, none identified thus far are likely to be orthologous to PWS-region SnoRNAs. 

In summary, the genes within the PWS-region primarily fall into three classes, ubiquitin ligases, RNA processing regulators and SnoRNAs. Genes in these classes regulate the expression of other genes, controlling rates of protein and RNA turnover, and influencing alternative splicing events, functions that are highly conserved in all vertebrate organisms. However, it appears that essentially all the genes in the PWS-region have arisen, and rapidly diverged, in the mammalian lineage. Consequently, although zebrafish have orthologs of the ancestral genes, they do not appear to have one-to-one orthologs of the PWS-region genes. This fact begs the question of how zebrafish could be used as models for PWS research if they don’t have PWS genes. The answer lies in the fact that dysregulation of PWS genes result in alterations in highly conserved developmental pathways, particularly evident in the hypothalamus. This is illustrated by studies of PWS-like syndromes, which implicate pathways that are highly conserved in humans, mice and zebrafish. 

### 2.2. PWS-Like Syndromes: 6q16 Deletions

Prader-Willi-like (PWL) syndromes share features of the PWS phenotype, however the genetic basis of these rare disorders differs. The implication is that the gene functions disrupted in PWL are likely to lie in genetic pathways that are important for the development of PWS phenotypes. By extension then, drugs targeting these pathways may prove effective for PWS patients. Elucidation of these conserved pathways and identification of potential drug compounds could greatly benefit from zebrafish models.

Although a variety of deletions have been reported to cause Prader-Willi related phenotypes, the most common PWL deletion is the 6q16 deletion comprising the bHLH-PAS family transcription factor *SIM1* [20,21,22]. *Sim1* heterozygous mice display hyperphagic obesity and reduced oxytocin expression in the hypothalamus [23,24]. Oxytocin neurons that project to the hindbrain have been implicated in satiety [25,26]. Recently, a small 6q16 deletion that results in PWL as been identified. The deletion leaves *SIM1* intact but deletes another gene, *POU3F2*, in the oxytocin developmental pathway [27]. Importantly, the entire *sim1, pou3f2, oxytocin* pathway has been shown to be conserved in human, mouse and zebrafish [27,28,29,30,31,32,33,34,35]. Together, these findings suggest a role for the oxytocinergic system in PWS symptoms and highlight the value of modeling phenotype in addition to genetic cause to uncover the developmental underpinnings of PWS.

### 2.3. Non Imprinted PWS Genes

Along with the imprinted PWS genes there are several non-imprinted genes located within the most common PWS chromosomal breakpoints. Four non imprinted genes, *NIPA1*, *NIPA2*, *CYFIP1* and *TUBGCP5*, are located upstream of *MKRN3*. These four genes lie between the two most common upstream PWS chromosomal breakpoints. Downstream of the PWS imprinted region are two paternally imprinted genes associated with Angelman Syndrome, *UBE3A* and *ATP10A*. Five non imprinted genes, *GABRB3*, *GABRA5*, *GABRG3*, *OCA* and *HERC2*, are then located before the most common downstream PWS breakpoint. Haploinsufficiency of these genes could modify the pathobiology of PWS in cases where they are deleted from the paternal chromosome.

Although all the non-imprinted genes appear to be important for neurological function, their potential contribution to PWS has not been well-studied. Interestingly, in contrast to the mammalian specific imprinted PWS genes, the Angelman Syndrome-related and the non imprinted PWS genes are highly conserved among vertebrates. These genes all have one-to-one orthologs between human and zebrafish with highly conserved synteny. It appears as though the mammalian imprinted PWS genes arose primarily by duplication and transposition of ancestral genes located elsewhere in the genome, accumulating smack in the middle of this highly conserved group of neural related genes. Therefore, it is tempting to speculate that these non imprinted genes influence PWS. Studies in zebrafish have high potential for shedding light on the function of these genes as their expression could be easily manipulated in this organism.

### 2.4. Mouse Models

In mice, the existence of a syntenic region containing orthologs of PWS-related imprinted genes has allowed for the generation of mouse models with large deletions spanning the entire PWS region in the hopes of recapitulating the complex phenotype of Prader-Willi patients. However, neonatal death in these models has precluded the study of the hyperphagia phenotype, which is a leading cause of morbidity in PWS. The first of these mutants harbored a deletion of the central portion of the paternally inherited chromosome 7, and a duplication of the maternally inherited copy. These mice began to fail and died within eight days after birth [36]. Similarly, in a large deletion of all imprinted genes in the PWS-associated region in mice, offspring failed to thrive and died within a week [37] (Table 3). Although these models come closest to replicating PWS in genotype, the severity of the resulting phenotypes prohibit their use for extensive study of the disorder. *Snrpn* deletions of varying sizes have been studied in an attempt to clarify the role of *SNRPN* in PWS, and locate the region of this gene that is critical for imprinting [38]. Several of these models in which much or all of the *Snrpn* sequence has been deleted have produced mice that fail to thrive and die early on, precluding phenotypic analysis. Some smaller deletion models in which only parts of the *Snrpn* gene have been removed showed improved survival, however they did not display observable abnormal phenotypes (Table 3). Zebrafish are not amenable to this analysis as they lack a comparable PWS imprinted region.

Single gene deletion models in mice have been more successful in achieving PWS-like phenotypes that can realistically be studied. Although these gene knockouts are much less extensive than the lack of expression of the paternal ch15q11–q13 region observed in PWS, the resulting mice display a variety of endophenotypes that are reminiscent of the disorder (Table 4). Unique to the *Magel2* knockout was impaired fertility and alterations in circadian rhythm, while the *Ndn* knockout mice suffered from respiratory distress [42,43,44]. Interestingly, the *Ndn* knockout had decreased oxytocin expression in the paraventricular nuclei of the hypothalamus [43]. *Magel2* and *Snord116* knockouts displayed growth retardation early in life, as is observed in PWS patients. In later life, the *Snord116* knockout model was hyperphagic, while the *Magel2* knockout had increased fat mass [45,46]. However, no model has recapitulated the concomitance of increased appetite and obesity, which is a prominent feature of PWS. A number of behavioral phenotypes have also been identified in the single-gene knockout models. The *Magel2* and *Snord116* knockouts had increased anxiety, and the *Ndn*-deficient mice showed improved spatial learning and memory as well as skin-scraping—a behavior commonly observed in PWS patients [43,44,45]. While these phenotypes are abnormal, the extent of their similarity to the cognitive, behavioral and psychiatric components of PWS remains unclear. 

While relevant endophenotypes appear in mouse models with partial genetic replication of PWS, models with full PWS deletions do not survive long enough to display the main characteristics of the disorder. This suggests that differences between mouse and human PWS-regions may preclude generation of mouse models that fully recapitulate PWS. On the other hand, single-gene knockout studies in mice have begun to uncover the role of several PWS-related genes. 

Zebrafish do not have one-to-one orthologs of the imprinted PWS-associated genes so direct PWS single gene knockout models cannot be generated in this organism. However, zebrafish do have orthologs of the ancestral precursors of the PWS genes. Studies of the ancestral genes in zebrafish may be highly informative for PWS pathology, as many of the PWS genes are members of multigene clusters with potential for redundant function. Because zebrafish often have a single ancestral gene, the effects of gene manipulation on neurodevelopment and function may be easier to discern. 

Genes that are outside of the PWS-region but result in PWS-like phenotypes, and are thus implicated in important pathways in PWS pathobiology, present additional fruitful avenues of research. For example, mutations in *SIM1*, leptin receptor (*LEPR*), pro-opiomelanocortin (*POMC*), melanocortin 4 receptor (*MC4R*), and more recently, *POU3F2* have all been associated with severe obesity, suggesting a convergence on the leptin-melanocortin pathway in association with oxytocin [25,27]. Mutations in zebrafish *lepr*, *mcr4*, and *pou3f2* have been generated, although few obesity-related studies have been performed as of yet. Zebrafish however, are powerful models for investigating molecular developmental pathways so we anticipate rapid progress in this area. Knowledge gained from these studies will likely reveal important new targets for pharmacotherapies.

Identifying drugs that could rescue PWS phenotypes would provide a promising way forward in developing curative therapies for this disorder. However, mice are impractical for large-scale drug discovery. On the other hand, models generated in zebrafish, either by gene-knockouts or mutagenesis screens, which display PWS-like endophenotypes, provide ideal drug discovery platforms. 

### 2.5. The Zebrafish Model: Conserved Neuroanatomy and Physiology

The last common ancestor of zebrafish and human was the lobe-finned fish. These fish were already highly sophisticated animals with extensive behavioral repertoires and exquisite ability to respond to environmental changes to maintain physiological homeostasis. Therefore, the human brain evolved upon this complex substrate resulting in the conservation of much of the underlying neuroendocrine and autonomic function. This observation gave rise to the idea of the primitive “reptilian brain”, an idea that was generally discarded when it became evident that the so-called primitive brain areas were extensively interconnected to higher cortical brain regions, and critically important for higher brain function. In fact, most neuromodulatory systems, which are critical targets of neuropsychiatric pharmacotherapies, arise from primitive brain areas. Conservation in primitive brain neuroanatomy and physiology is apparent in the neuroendocrine hypothalamus, and thus, has important implications for the usefulness of the zebrafish model for PWS research.

Many of the characteristics associated with PWS, including hyperphagia, temperature instability, high pain threshold, sleep-disordered breathing, and endocrine abnormalities such as growth hormone deficiency, central adrenal insufficiency, central hypothyroidism, and hypogonadism, are linked to hypothalamo-pituitary axis (HPA) dysfunction [1,47]. The evolutionarily conserved HPA, consisting of the hypothalamus and pituitary gland (hypophysis), serves as the connection between the central nervous system (CNS) and the endocrine system, which together regulate whole-body homeostasis and reproduction [48].

The mammalian neuroendocrine hypothalamus is composed of several nuclei that contain distinct clusters of neurons that project to the pituitary. The magnocellular system consists of large AVP and OXT producing neurons located in the supraoptic nucleus (SON) and paraventricular nucleus (PVN) that project to neurohemal sites in the posterior pituitary (neurohypophysis) where these hormones are released directly into the systemic circulatory system. The parvocellular system consists of smaller neurons that control the anterior pituitary (adenohypophysis). The major nuclei of the parvocellular system are the PVN, arcuate nucleus (AR), anterior periventricular nuclei (aPV) and preoptic area (POA). Parvocellular neurons from these nuclei project to a small portal blood system releasing one or more of the following six hormones, thyrotropin releasing hormone (TRH), corticotropin releasing hormone (CRH), growth hormone-releasing hormone (GHRH), somatostatin (SST), gonadotropin-releasing hormone (GnRH), and dopamine (DA). These hormones each initiate the release of distinct hormones from the adenohypophysis into the blood stream. 

The nuclei of the hypothalamus are extensively interconnected and receive diverse input from multiple CNS regions. In addition, these cells have extensive extrahypophysial projections, releasing their peptide hormones as modulatory neurotransmitters within the brain, presumably to coordinate behavior with maintenance of physiological homeostasis. This Gordian entanglement of neuronal circuitry likely ensures the exquisite control of physiologic homeostasis that is achieved by the hypothalamus. Understanding how the highly complex chemoarchitecture and circuitry of the hypothalamus coordinates brain-body communication presents an exciting challenge, and is not only important for identifying potential therapeutics for PWS, but will contribute to a wide range of neuropsychiatric conditions, such as autism, obesity, anorexia, affective, obsessive-compulsive, and anxiety disorders [49]. The multifaceted experimental advantages of zebrafish for genetic and pharmacological analysis, visualizing and manipulating brain circuits, and behavioral analyses will help meet this challenge. 

A fundamental tenet of evolutionary biology is that critical structures are not reinvented, instead they are built upon [50,51]. What this means for comparison between fish and humans is that the “primitive brain”, representing the brain of the last common ancestor of teleosts and mammals, should largely be the same, particularly for core functions. Layered on top of the core functions would be extensive adaptations acquired during the incredible brain expansion that defines the path of human evolution. This is illustrated by the extensive reciprocal connectivity between the higher cortical brain regions and the hypothalamic nuclei. As the hypothalamus and other brain stem regions were already highly complex and absolutely required for survival, the underlying organization and functions of these regions would be highly conserved. On the other hand, each species must adapt to its specific environmental pressures. Therefore, one finds, as expected, specific differences between species. Along these lines, an expanding use of the zebrafish model is to help differentiate between core functions and species specific adaptations when evaluating mouse studies. If a process can be shown to occur the same way in zebrafish and mice, it is likely to be a core function, and therefore, also function in humans [27,52]. 

Extensive studies over the past 15 years have demonstrated remarkable conservation of brain architecture, neuroendocrine cell type, function, and molecular control of hypothalamic development between zebrafish and mammals. These studies have been recently reviewed [8,48]. In short, zebrafish have similar neuroendocrine cells producing OXT, AVP, TRH, CRH, GHRH, SST, GnRH, and DA as are seen in mammals. These neurons project similarly to the pituitary where they control the release of the same pituitary hormones as seen in mammals. In addition to the neuroendocrine cells, many other hypothalamic peptidergic neurons have been characterized in zebrafish including, melanocortin, hypocretin, and somatostatin [53,54,55] Remarkably, the functions and regulation of the various endocrine axes are largely the same, taking into consideration obvious lifestyle differences. Moreover, many aspects of hypothalamic circuitry are conserved. For example, recent data suggests that a key part of the essential hypothalamic circuit controlling energy homeostasis in mammals, the melanocortin system, is conserved zebrafish [8]. Overall, zebrafish appear well suited for modeling PWS, at least based on hypothalamic endophenotypes.

### 2.6. Technologies for Phenotypic Analysis: PWS Endophenotypes

A variety of strategies for phenotypic analysis in zebrafish enable the study of PWS endophenotypes resulting from genetic or pharmacologic manipulations. These include ‘omics analyses, such as RNAseq transcriptomics, quantification of fluorescence or luciferase in transgenic reporter fish, analysis of morphology using high-throughput hyperdimensional screens, and even behavioral assays [56,57,58,59,60,61,62,63,64,65,66,67,68,69]. 

Perhaps the most well documented endophenotypes in PWS are deficits in hypothalamic development and function. The numbers of oxytocin producing neurons, as well as circulating levels of oxytocin peptide are decreased in PWS patients. GH production is likewise reduced. In fact, GH replacement is one of the only therapies shown to improve PWS symptoms. GnRH, follicle-stimulating hormone (FSH) and luteinizing hormone (LH) perturbations are also strongly implicated in PWS. Hypocretin is involved in promoting wakefulness and regulating appetite, and the melanocortin pathway is critical for appetite control and energy expenditure. Given the high evolutionary conservation of the hypothalamus, this brain region may provide an especially fertile substrate for developing zebrafish models of PWS. Therefore, the following discussion mostly focuses on hypothalamus based endophenotypes.

Transgenic zebrafish are well suited to the detection of abnormalities in the development and regulation of neuronal populations. Especially relevant to PWS are the hypothalamic nuclei, for which several transgenic zebrafish models already exist. Zebrafish larvae expressing GFP driven by an oxytocin promoter have been used to study the effect of fetal ethanol exposure on brain development, and as a model for fetal alcohol syndrome [70]. Also, POMC producing cells, an important component of the HPA, have been tagged in transgenic zebrafish lines [71,72]. In addition, zebrafish have been generated that express fluorescent proteins in GnRH, FSH, LH, and prolactin (PRL) secreting cells, which are deficient in PWS [73,74]. Likewise, hypocretin transgenic lines have been developed and effectively used for opticogenomic studies of wakefulness in zebrafish [75]. Transgenic models can be used to investigate molecular pathways involved in hypothalamic function and to discover drugs that effect endocrine output. A recent study screened zebrafish with fluorescently marked pancreatic β-cells using automated reporter quantification *in vivo* (ARQ-iv) combined with robotics. In this study, 24 FDA-approved drug candidates were identified that modulated β-cell numbers [68]. Conceptually similar strategies may prove highly productive for identifying potential pharmacotherapeutics for PWS. Although there are several transgenic lines that would potentially be useful for high throughput drug screening, new lines specifically designed for this purpose might be more productive in the end. As transgenic zebrafish are relatively easy to make, optimized designs should be pursued. 

Zebrafish morphology is another potential output in PWS-related mutational or drug screening. Abnormalities in growth and body fat can be easily quantified in fish. In studies on body fat accumulation, anesthetized fish were weighed, and their body fat was quantified by computed tomography. The ratio of body fat to weight provided a measure of obesity across treatment groups [76]. Developmental deformities also represent relevant PWS endophenotypes. However, zebrafish morphology has been a problematic output for high-throughput screens since it is typically assessed by non-quantitative means. Subjective assessment of morphology may not be sensitive enough to detect minor defects in tissue architecture. Recently, however, an automated phenomics technique has been developed for high-throughput screens based on vertebrate morphology. Optical projection tomography (OPT) is capable of quickly generating and analyzing three-dimensional images of zebrafish embryos using an automated fluidics-based system. Proof of concept was illustrated by the characterization of craniofacial abnormalities in response to teratogenic chemicals [66]. As morphology screens continue to advance in sophistication, they will become valuable tools for analysis of zebrafish models of PWS resulting from genetic manipulation, and may provide informative readouts for large-scale drug screening. 

Behavioral problems are a particularly challenging aspect of PWS. A variety of behavioral traits can be rapidly assessed in the zebrafish model, which has already been used to study neuropsychiatric and drug-related disorders [77,78]. In a recent study, recordings of zebrafish larvae placed in multilane plates facilitated the assessment of responses to a visual stimulus displayed on a screen. Assessed responses include avoidance behavior, thigmotaxis as measured by preference for the edge of the lane, swim speed, resting behavior, and social distance between larvae [57,79]. Optogenetic manipulation now provides direct control over behavioral circuits [75,80]. Social behavior can be further investigated by preference of adult zebrafish for the conspecific portion of a tank containing a live or virtual zebrafish, as compared with an empty sector. Fear can be measured by avoidance of the Indian leaf fish, a known zebrafish predator. Preference for the bottom of the tank, diving behavior, and preference for the dark portion of a tank, all measured by video cameras, have been used as measures of fish anxiety. Cognitive tasks can be developed similarly to mouse assays with the use of T-maze-shaped tanks. Activity monitoring can also be used to measure circadian rhythms and habituation [77]. Even food seeking and digestive physiology can be directly measured [81]. Food-seeking, tantrums, and skin-picking are all problematic PWS-associated behaviors for which no effective drug treatment has been routinely helpful. Additionally, PWS is often accompanied by mood disorders. The ability to rapidly quantify behavior in zebrafish with high sample size provides an array of additional phenotyping options for PWS models in this organism.

### 2.7. Strategies for Targeted Gene Manipulation

Reverse genetics in zebrafish is a promising approach to examine the complex range of phenotypes associated with PWS. One strategy is to disrupt orthologs of PWS-associated genes, and study the resulting phenotypes. As zebrafish have orthologs of the ancestral PWS-related genes, often in a much simplified context (e.g., a single MAGE gene compared with 50 human genes), zebrafish may inform the basic function of these complex genes. In addition, the contribution of the non-imprinted genes in the PWS region could be investigated. Beyond simple gene mutations, the ease of genetic manipulation makes zebrafish an ideal model for the study of gene function by knock-out, knock-in, over/mis expression, and knock-down [82]. 

A majority of zebrafish genes have already been mutated through the efforts of several large mutagenesis programs. The Zebrafish Mutation Project (ZMP), at the Sanger Institute identified mutant genes by Targeting Induced Local Lesions in Genomes (TILLING) libraries of ENU induced mutations. These mutant fish are available from the Zebrafish International Resource Center (ZIRC) and/or European Zebrafish Resource Center (EZRC). Another large scale genome wide screen has been done using insertional mutagenesis, and these fish are available from ZIRC [83]. In addition to these large scale genome wide collections, many labs have been generating and identifying mutations for the past twenty years, most all of which are available from the resource centers or from individual labs. Zebrafish investigators are traditionally quite open and generous. 

To create new mutant lines, genome editing has been routinely accomplished using transcription activator-like effector nucleases (TALENs), and can now be achieved with greater efficiency using clustered regularly interspaced short palindromic repeats (CRISPR) technology. While TALENs require protein engineering and are time-consuming, they can be designed to specifically target any region of a gene of interest. Although somewhat more limited by sequence requirements, the CRISPR system can introduce specific mutations quickly through alteration of the sequence of the guide RNA associated with the Cas9 enzyme. This strategy facilitates the study of neurological phenotypes with a selected genetic basis [77]. For example, CRISPR-mediated knockdown of carbonic anhydrase related proteins (CARPs) that are highly expressed in the CNS has revealed a role in regulation of movement. Abnormal movement patterns were detected by video tracking of zebrafish embryos [84]. Importantly, the knock-in approach in which exogenous DNA is incorporated into the genome is also possible with CRISPR technology. This strategy has already been used to insert transgenic markers, loxP sites and small affinity tags such as HA at specific loci in zebrafish [85,86,87]. Additionally, single nucleotide polymorphisms have been rescued by CRISPR-mediated homology directed repair [88]. The incorporation of DNA sequences with disease-specific SNPs has been accomplished in mouse and cell culture, suggesting that this approach to the study of genetic disorders is possible in zebrafish as well. 

Mutations are often associated with compensatory events that counteract the effect of the lesion [89]. Antisense morpholino oligonucleotides (MOs) do not trigger the same compensatory mechanisms, so they provide a complement to genome editing and can be used to modulate gene expression. On the other hand, MOs are subject to significant off target effects, so MO experiments must be well-controlled and interpreted with caution [90,91]. Translation blocking MOs bind near the AUG start codon of target transcripts, thereby blocking ribosome entry and translation initiation. Alternatively, splice blocking MOs can be designed to bind to splice donor or acceptor sites to prevent normal splicing, often resulting in nonsense transcripts that are rapidly degraded in the embryo. Morpholinos enable rapid targeting of PWS-associated genes, as well as genes associated with hypothalamic development and regulation, resulting in endophenotypes relevant to PWS. Several studies have used these techniques to investigate genes implicated in PWS-like disorders demonstrating a startlingly high degree of conservation in the molecular control of brain development [27,28,30,31,92,93,94]. Although MOs are an important tool for investigating early brain development, they become essentially inactive by four to five days post-fertilization, making them less useful for behavioral analyses.

### 2.8. Approaches for Gene Discovery in Zebrafish

Zebrafish were originally selected for development as a model organism based on the ability to efficiently perform genetic analyses in this organism [95]. In the past, mutagenesis screens focused on embryonic development where they have had a major impact on our understanding of vertebrate development. More recently, mutagenesis screens have been utilized to study myriad aspects of biology. The discovery of disease genes is rapidly becoming a major focus for mutagenesis screens. Several approaches have been utilized including chemical mutagenesis, insertional mutagenesis, and protein trapping [96,97,98,99,100,101,102]. The standard technique for chemical mutagenesis involves exposure to ethylnitrosourea (ENU), which induces point mutations in the germline. Although highly efficient, the random nature of this technique complicates location of the mutation and identification of the mutated gene. Insertional mutagenesis involves the incorporation of exogenous DNA by injection of a retrovirus or transposon. In contrast to the ENU approach, mutated genes can be rapidly identified due to the inserted DNA, however, the mutagenesis throughput is much smaller. Similar ease of identification is possible using protein-trapping techniques, in which integration of a splice acceptor into a transcriptionally active locus results in a truncated protein and expression of the reporter construct. Throughput levels are comparable with other insertional strategies [102]. Mutagenesis has already yielded zebrafish models for a variety of CNS disorders, including Alzheimer’s disease, Dravet Syndrome, Schizophrenia and addiction [79,103,104]. A few of these models have been further utilized for drug screening, identifying promising new pharmacotherapies for their targeted disorders [58,79]. This suggests that forward genetics can also generate PWS-associated endophenotypes with an identifiable genetic basis that can be rescued in large-scale drug screens to identify therapeutic targets.

### 2.9. Pharmacogenomic Methods in Zebrafish

Zebrafish are increasingly being utilized for drug discovery and toxicological studies due to their small size and the high concordance of drug response between this organism and mammalian systems. In addition, dosing is straightforward as compounds are simply added to the embryo water. A major advantage of zebrafish derives from the fact that bioactive compounds can be identified based on their effects on embryonic phenotype [64,105]. Therefore, no *a priori* knowledge of disease genes or pathways is required for identifying bioactive compounds. Likewise, phenotype based screens are agnostic to target organ, meaning that a drug may target a seemingly unrelated process but it will still be identified in these screens. For identifying compounds that effect hypothalamic development and function, as could be done for PWS endophenotypes, this facet of zebrafish phenotype screening would be particularly advantages, since the neuroendocrine system is regulated by myriad, complex, poorly understood, feedback mechanisms.

A variety of chemical screen designs have been utilized over the past 15 years since the first demonstration of this approach in zebrafish. Screens have utilized numerous phenotypic endpoints including: developmental morphology, whole-mount *in situ* hybridization (WISH) for mRNA expression patterns, whole-mount immunohistochemistry (IHC) for protein expression patterns, survival, apoptosis, and behavior [63,64,68,69,79,105,106,107,108,109,110,111,112,113,114,115,116,117,118,119,120,121,122,123,124,125]. Several different compound collections have been utilized for zebrafish screening projects ranging from highly diverse chemical backbone libraries to FDA approved drug collections. For projects aiming to identify potential chemical backbones for developing new drug classes, libraries consisting of a high diversity of quality drug-like and lead-like compounds have been used, for example, Chembridge DIVERSet-EXP collection of synthetic compounds. Alternatively, if the goal is to identify genetic pathways and pharmacological targets involved in the disease process, then libraries of highly characterized compounds, such as The Library of Pharmacologically Active Compounds (LOPAC) collection of 1280 well-characterized pharmacologically active compounds, or, collections such as MicroSource spectrum, which contains 1040 US clinical trial stage drugs, 240 additional drugs marketed internationally and 800 natural products, could be used. More recently, repurposing FDA approved drugs and compounds in clinical trials has been the focus of several screens. Compound libraries for repurposing include collections such as: Enzo SCREEN-WELL^®^ FDA approved drug library V2; the Johns Hopkins Drug Library (JHDL) consisting of 2290 drugs approved for use in humans by the FDA or international counterparts, 775 drugs at various stages in clinical trials, and 66 rare drug compounds; and the National Center for Advancing Translational Sciences (NCATS) collection of 2500 small molecular entities approved for clinical use by U.S., European Union, Japanese and Canadian authorities, along with about 1000 additional investigational compounds [64,68,105,107]. 

### 2.10. Fast Track to PWS Pharmacotheraputics

According to the NCATS, discovering new uses for approved drugs provides the quickest possible transition from bench to bedside. The recent success of using zebrafish for phenotypic screens, particularly for repurposing FDA approved drugs presents an opportunity for fast track identification of potential pharmacotherapeutics for PWS. Perhaps the simplest approach would be to use changes in PWS-relevant hypothalamic hormone producing cells types as endpoint endophenotypes. For example, one could assay for FDA approved compounds that are capable of increasing fluorescence of *oxytocin:EGFP* transgenic embryos, indicating either an increase in the number of oxytocin expressing cells or an increase in oxytocin expression. The same could be done using GH, FSH or LH, all of which are deficient in PWS patients. The power of the screen would likely benefit from using several of these assays. This approach of simply screening zebrafish embryos for compounds that increase numbers or expression of normal cell types, which are relevant to adult disease states, has recently proved to be highly successful [68,118]. A further step could be to perform these screens using zebrafish with PWS-related genetic deficits, such as *sim1* mutant embryos. The endpoint would then be to rescue the endocrine defects in the mutant embryos. The power of these phenotypic screens derives from the fact that they are molecular target blind and tissue agnostic so it doesn’t matter if the reduction in oxytocin expression is a direct result of PWS pathology or if oxytocin is unrelated to PWS symptoms. If a compound increases oxytocin in zebrafish embryos, the chances are good that it will also do so in people, regardless of whether the mechanism is targeted or pleiotropic. Such bioactivity has a reasonable chance of helping to alleviate PWS symptoms. Since the screens are performed using FDA approved drugs, identified compounds could be immediately used by PWS patients. 

## 3. Conclusions

Although the genetic basis of PWS is known, how the PWS-associated chromosomal anomalies drive neurodevelopmental deficiencies underlying this disorder is poorly understood. Mouse models have been generated that closely recapitulate PWS genetic defects, however, differences between mouse and humans appears to preclude phenocopy of this disorder between the species. On the other hand, the majority of PWS-related genes are highly conserved in mammals so a number of mouse models are providing important insight into the genetic underpinnings of PWS. Examination of potentially orthologous genes in zebrafish indicates that the imprinted PWS region has newly arisen in the mammalian lineage, within a highly conserved cluster of non-imprinted neural related genes. Thus, zebrafish models of PWS genes would focus on the ancestral precursors of the imprinted genes or on the non-imprinted genes. In contrast, the genes and pathways controlling development, which are presumably disrupted in PWS, are highly conserved and thus can be productively modeled in zebrafish. This is particularly true in the hypothalamus where neuroendocrine cell types that are effected in PWS are highly conserved. Therefore, forward and reverse genetic approaches in zebrafish accompanied by phenotypic analysis technologies can be used to identify drug targets and lead to a deeper understanding of the pathways underlying PWS-associated phenotypes, complimenting ongoing studies in mice. An important advantage of zebrafish is that potential PWS models can be utilized for high-throughput drug screening, a feature not found in any other vertebrate model system. Given our current understanding of the molecular genetic underpinnings of PWS and the long lead times for drug development, a phenotypic screening program for FDA approved drugs in zebrafish may represent the fastest path to identifying potential curative therapies. 

## Figures and Tables

**Table 1:** Medical characteristics and treatment options.

Medical Symptom	Age of Onset	Treatment Options
hypotonia and feeding difficulties	0–9 months	feeding assistance, nasogastric tubes
hyperphagia	4.5–8 years	behavioral therapy
short stature	puberty	GH therapy, allows patients to reach full adult height
hypoplastic genitalia	birth	hormonal replacement therapy
dysmorphic features	birth	none
sleep-disordered breathing and daytime hypersomnolence	childhood to adolescence (Nixon and Brouillette)	adenotonsillectomy, nocturnal ventilation, weight control, and behavioral interventions
cognitive disability	childhood	none
skin picking and obsessive behavior	5 years	behavioral therapy
oppositional behavior and tantrums	5 years	behavioral therapy and psychiatric drugs

**Table 2:** Mouse and zebrafish PWS-related genes.

Human	Mouse	% aa	Zebrafish	% aa	Chr.
*FRAT1*; Chr10 *	*Peg12*	70	*gbp* ^+^	63	16
*MKRN3*	*Mkrn3*	63	*mkrn1*	48	4
*MAGEL2*	*Magel2*	53	*ndnl2* ^&,+^	48	23
*NDN*	*Ndn*	82	*ndnl2* ^&,+^	40	23
*NPAP1*	*Pom121*; Chr5 *	28	*pom121* ^+^	24	10
*SNRPN*	*Snrpn*	100	*snrpb*	93	6
*SNORD107*	*Snord 107*		-		
*SNORD64*	*Snord 64*		-		
*SNORD116*@27	*Snord116*@27		-		
*SNORD115*@41	*Snord115*@130		-		
*SNORD108*	-		-		
*SNORD109A*	-		-		
*SNORD109B*	-		-		

% aa, percent amino acid identity to human; Chr., Chromosome; *, ortholog is located outside the PWS-associated region; -, no identified orthologous gene; ^&^, *ndnl2* is orthologous to both *MAGEL2* and *NDN*; ^+^, zebrafish mutant has been generated; @, copy numbers according to [11].

**Table 3:** Phenotypic characteristics of chromosomal deletion mouse models of PWS.

Failure to Thrive/Early Fatality	No Observable Abnormal Phenotype
Maternal duplication and paternal deletion in PWS region [36]	Deletion of exon 1 of Snrpn [39]
6.8 Mb deletion spanning PWS and AS regions [37]	Deletion of *Snrpn* exon 2 [40]
Deletion spanning from *Snrpn* to *Ube3a* [40]	Double deletion of *Snrpn* exon 2 and *Ube*3a [40]
Deletion of *Snrpn* exons 1–6 and distal portion of IC [41]	Deletion of *Snrpn* exon 6, parts of exons 5 and 7 [41]
4.8 kB deletion removing exon 1 of *Snrpn* and most of the differentially methylated region 1 (DMR1) [39]	

**Table 4:** Phenotypic characteristics of single gene or gene cluster deletion mouse models of PWS.

Endo-Phenotype	*Ndn* [43]	*Magel2* [42,44,46]	*Snord116* [45]
Reduced oxytocin expression	Yes	-	-
Postnatal growth retardation	X	Yes	Yes
Increased anxiety	-	Yes	Yes
Respiratory distress	Yes	-	-
Skin scraping	Yes	-	-
Improved spatial memory and learning	Yes	X	X
Altered circadian output	-	Yes	-
Impaired fertility	X	Yes	X
Increased fat mass	X	Yes	X
Hyperphagia	-	X	Yes

Yes, phenotype was present; X, phenotype was absent; -, phenotype was not assessed.
